# Personalised viscoelastometry-guided systemic thrombolysis for high- and intermediate-high-risk acute pulmonary embolism in the ICU: a single-centre randomised controlled interventional feasibility trial

**DOI:** 10.1186/s40635-026-00903-7

**Published:** 2026-04-29

**Authors:** András Kállai, Anna Párkányi, Máté Berczi, Dalma Skultéti, János Domonkos Stubnya, Hanna Dóra Szász, Gergely Szombath, Adrienne Fehér, Zsolt Dániel Iványi, János Gál, János Fazakas

**Affiliations:** 1 Department of Anaesthesiology and Intensive Therapy, Semmelweis University, Üllői Street 78/B, Budapest, H-1082 Hungary; 2 Pediatric Centre, Division of Neonatology, Semmelweis University, Budapest, Hungary; 3 Department of Central Anesthesiology and Intensive Therapy, St. John’s Central Hospital of North Buda, Budapest, Hungary; 4 Department of Internal Medicine and Haematology, Semmelweis University, Budapest, Hungary; 5 Department of Laboratory Medicine, Semmelweis University, Budapest, Hungary

**Keywords:** Feasibility study, Precision medicine, Point-of-care blood coagulation tests, Pulmonary embolism, Thrombolytic therapy

## Abstract

**Background:**

Systemic thrombolysis is recommended for high-risk pulmonary embolism, but is associated with a substantial bleeding risk due to thrombolysis-induced coagulopathy. However, recent advances in precision and personalised care offer the potential to reduce mortality as well as the incidence of chronic thromboembolic pulmonary hypertension and post-pulmonary embolism impairment, thereby improving long-term outcomes.

Our primary objective was to evaluate the feasibility of a viscoelastic testing-guided, low-dose, prolonged systemic thrombolysis protocol in acute pulmonary embolism. Exploratory secondary objectives included assessment of the safety and efficacy of the protocol using an individualised approach tailored to each patient’s coagulation profile.

**Methods:**

This single-centre, prospective, randomised interventional feasibility trial was conducted at Semmelweis University (Budapest, Hungary). Adult pulmonary embolism patients were randomly assigned to a control group (CG) receiving the ESC-recommended 100 mg/2 h dose of rtPA or a viscoelastometry-guided group (VGG), in which the rtPA dose and duration were adjusted based on viscoelastic tests. Transthoracic echocardiography (TTE) was performed every two hours to monitor right ventricular (RV) function and determine cessation of systemic thrombolysis in VGG. The primary outcome was feasibility; the secondary outcome was safety and efficacy.

**Results:**

Among 33 enrolled patients, 19 were included in the analysis (CG: 7**;** VGG: 12). Patients in the VGG received significantly lower doses of rtPA (median 32.00 mg [IQR 20.95–42.75 mg]) despite longer infusion duration (median 8.5 h [IQR 6.6–10.0]) and showed no coagulopathy. RV dysfunction persisted in two CG patients, whereas in the VGG it resolved in all patients. Major bleeding occurred in two patients in the CG and in one patient in the VGG.

**Conclusions:**

Viscoelastometry-guided, individualised, low-dose systemic thrombolysis is feasible and supports a personalised approach to thrombolysis in the management of pulmonary embolism.

**Supplementary Information:**

The online version contains supplementary material available at 10.1186/s40635-026-00903-7.

## Introduction

### Background and rationale

Acute pulmonary embolism (PE) is the third leading cause of cardiovascular mortality behind myocardial infarction and stroke, with an estimated annual incidence of 39–115 cases per 100,000 population [1]. Due to the substantial burden and associated mortality, the 2019 European Society of Cardiology (ESC) guidelines recommend systemic thrombolysis for patients with high-risk PE [2]. Indeed, the absence of treatment may result in mortality rates approaching 70% in high-risk PE, and 35% in intermediate-high risk cases. Appropriate therapy, particularly systemic thrombolysis, can reduce mortality by more than 50%, underscoring the importance of timely and targeted intervention [3, 4]. However, thrombolysis carries significant risks, with major bleeding in 13.8% of cases, and intracranial haemorrhage in 3.8% of cases. Moreover, thrombolysis failure occurs in 8.2% of patients, defined as persistent haemodynamic instability or ongoing right ventricular (RV) dysfunction [5, 6].

Systemic thrombolysis with recombinant tissue plasminogen activator (rtPA) induces targeted fibrinolysis by converting plasminogen to plasmin at fibrin-binding sites, resulting in clot dissolution. However, rtPA may also interact with circulating fibrinogen—especially at higher doses—leading to significant fibrinogen depletion. In addition, the generation of high levels of fibrin degradation products (FDPs) further contributes to thrombolysis-induced coagulopathy, thereby disrupting haemostatic balance [7].

PE management is guided by early mortality risk and includes anticoagulation, systemic thrombolysis, catheter-directed or ultrasound-enhanced thrombolysis, and surgical thrombectomy. Systemic thrombolysis is the first-line treatment for high-risk PE, enabling rapid haemodynamic stabilisation and reperfusion. As alternative strategies lack sufficient evidence for routine use, systemic thrombolysis remains the standard of care [8]. According to ESC guidelines, standard thrombolysis consists of 100 mg rtPA administered over 2 h [2].

Several studies have investigated low-dose systemic thrombolysis (25–50 mg rtPA) to reduce bleeding risk and mortality; however, none incorporated repeated viscoelastic testing or frequent echocardiographic monitoring as applied in our study [9–17]. Recent research underscores the need for safer, more effective thrombolytic strategies in high- and intermediate-high-risk PE [18]. In line with personalised medicine, these findings support the consideration of tailored, reduced-dose, prolonged systemic thrombolysis to optimise outcomes while minimising adverse events [19].

### Objectives

These considerations led us to examine the feasibility of individualised low-dose, prolonged systemic thrombolysis to improve safety and efficacy, while broadening treatment eligibility. We hypothesised that reduced rtPA dosing could mitigate thrombolysis-induced coagulopathy, lower the risk of major bleeding, and allow both high- and intermediate-high-risk PE patients to benefit from thrombolysis, thereby improving long-term outcomes and quality of life.

The study aimed to enable close clinical and laboratory monitoring to detect and prevent therapy-related coagulopathy. Serial viscoelastic testing and repeated echocardiographic assessment of right ventricular function guided real-time therapeutic adjustments in the intervention group.

## Methods

### Study design

This single-centre, randomised, controlled interventional study was conducted at the Central ICU of the Department of Anaesthesiology and Intensive Therapy, Semmelweis University (Budapest, Hungary), as a feasibility study (ClinicalTrials Identifier: NCT06667882), and is reported in accordance with the CONSORT 2025 guideline [20].

### Ethical considerations

This study complied with the Declaration of Helsinki and the ICMJE recommendations. Ethics approval was granted by the Hungarian National Public Health Centre (65187–5/2021/EÜIG). Written informed consent was obtained from all participants. All procedures respected the participants’ privacy rights.

### Eligibility criteria

All patients admitted to the ICU with acute pulmonary embolism confirmed by computed tomographic pulmonary angiography (CTPA) were screened for eligibility. Patients classified as having high or intermediate-high early mortality risk according to the 2019 ESC guidelines were included in the final analysis (see *Table S1* in the *Supplementary Methods* for the classification). Exclusion criteria included low or intermediate-low mortality risk, contraindications to systemic thrombolysis according to ESC guidelines (see *Table S2* in the *Supplementary Methods*), or lack of informed consent [2].

### Interventions

Following CTPA-confirmed PE, eligible patients were admitted to the ICU for systemic thrombolysis with continuous monitoring (blood pressure, ECG, pulse oximetry). The indication for thrombolysis was jointly approved by two intensive care specialists. Hourly blood samples were collected via a radial arterial cannula into 3.5-mL tubes containing 3.2% sodium citrate (Vacuette®, Greiner Bio-One International GmbH, Kremsmünster, Austria) and immediately analysed using the ClotPro® system (Haemonetics Corporation, Enicor GmbH, Munich, Germany). An independent assessment of measurement variability for the ClotPro viscoelastic assays was not performed, as measurements were conducted once per time point in accordance with the study protocol. The ClotPro system is CE-certified, and its analytical performance, including assay variability, has been established during regulatory validation in accordance with predefined acceptance criteria.

To avoid the anticoagulant effect of heparin on blood sampling, the arterial line was flushed with heparin-free 0.9% saline. Arterial blood gas (ABG) analyses were performed every two hours to monitor respiratory/metabolic status and detect drops in haemoglobin levels. To minimise the risk of bleeding complications, only essential arterial and peripheral venous cannulation was performed; central venous and urinary catheters were placed only when necessary.

Before systemic thrombolysis, baseline 2D transthoracic echocardiography (TTE) was performed in all patients to assess RV size and function, including intraventricular septal configuration (D-shape), RV dimensions, RV/LV ratio, tricuspid annular plane systolic excursion, pulmonary acceleration time, stroke volume index, eccentricity index, and mitral inflow pattern. All examinations were performed using a Philips CX50 device (Philips Ultrasound Inc., Bothell, WA, USA) by a single expert (A.K.) to minimise interobserver variability.

If severe bleeding or adverse symptoms (e.g. chest pain) occurred during the procedure, thrombolysis was immediately discontinued.

#### Interventions in the control group

In the CG, treatment followed the 2019 ESC guideline, with 100 mg rtPA (Alteplase, Actilyse®, Boehringer Ingelheim, Biberach an der Riß, Germany) administered over 2 h. Viscoelastic tests (EX-, FIB-, TPA-, RVV-, ECA- and AP-tests) were performed hourly. TTE was repeated after thrombolysis. In selected patients, additional ECA tests were conducted at 2 and 10 min, and ECA-, EX-, FIB- and AP-tests at 20 min post-thrombolysis.

#### Interventions in the viscoelastometry-guided group

**Fig. 1:**
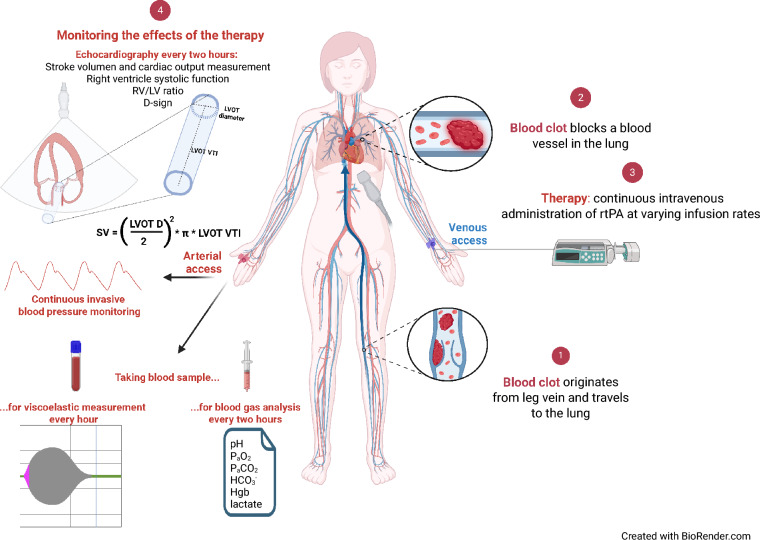
Methodological framework of viscoelastometry-guided thrombolysis: (1) clot formation: thrombus development in the deep veins of the lower limbs. (2) Embolisation: migration of the clot to the pulmonary artery causing pulmonary embolism (PE). (3) Initiation of thrombolysis: continuous recombinant tissue plasminogen activator (rtPA) infusion via peripheral access following PE confirmation. (4) Therapeutic monitoring: transthoracic echocardiography (TTE) every two hours to assess right ventricular function. Invasive arterial blood pressure monitoring. Hourly VET for dose titration to optimise efficacy and minimise bleeding risk. Arterial blood gas analysis every two hours to assess oxygenation, acid–base balance, and haemoglobin level. (5) Cessation of thrombolysis: discontinuation upon resolution of the D-sign

In the VGG, an extended-duration, viscoelastometry-guided and echocardiography-controlled protocol was applied, using low-dose rtPA with dynamic dose adjustment (Fig. 1*).* Viscoelastic tests (EX-, FIB-, TPA-, RVV-, ECA- and AP-tests) were performed hourly. The rtPA dose was increased when fibrinolysis was insufficient on EX and ECA tests, and reduced when excessive fibrinolysis was detected on the FIB test. If maximum lysis (ML) was < 50% on EX and ECA tests, the rtPA dose was increased. During systemic thrombolysis, if FIB MCF decreased by more than 15% between two measurements, the rtPA dose was reduced. Fibrinogen supplementation was administered whenever FIB maximum clot firmness (MCF) dropped below 9 mm. TTE was repeated every two hours to determine intervention cessation upon D-shape resolution. In selected patients, additional ECA tests were performed at 2 and 10 min and ECA-, EX-, FIB- and AP-tests at 20 min post-thrombolysis.

The rtPA infusion adjustments were made only with the consensus of two intensivists trained in viscoelastometry.

### Outcomes

The primary outcome was feasibility, defined as adherence to the study protocol. This was assessed by the proportion of scheduled procedures completed for each patient, including arterial blood sampling, viscoelastometry measurements, rtPA infusion adjustments, and echocardiographic assessments. Protocol adherence was expressed as the percentage of completed procedures across the study population.

Feasibility thresholds were defined using a structured progression-criteria framework commonly applied in pilot and feasibility studies to guide decisions on whether to proceed to a definitive trial. Consistent with published recommendations that advocate the use of a traffic-light or stop–amend–go approach for defining feasibility outcomes in pilot trials, protocol adherence ≥ 80% was considered indicative of acceptable feasibility to progress without changes, adherence between 70 and 79% was considered feasible with modifications, and adherence < 70% was considered not feasible without major protocol redesign [21, 22]. These thresholds were intended to guide feasibility decision-making rather than to serve as efficacy benchmarks.

Secondary outcomes included an exploratory comparison of safety and efficacy between the groups, evaluated by therapy-related mortality, major bleeding, thrombolysis-induced coagulopathy and resolution of RV dysfunction.

Therapy-related mortality was defined as death attributable to thrombolysis-related complications, primarily fatal bleeding events or unresolved RV dysfunction despite thrombolysis, occurring during treatment or within 30 days thereafter. Deaths clearly attributable to pre-existing comorbid conditions were not classified as therapy-related mortality. Major bleeding was defined according to ISTH criteria as fatal bleeding, bleeding in a critical area or organ, a haemoglobin drop ≥ 2 g/dL, or transfusion of ≥ 2 units of blood [23]. Thrombolysis-induced coagulopathy was defined as post-thrombolysis MCF values falling below the assay-specific lower limit of normal.

RV dysfunction was defined according to the European Society of Cardiology guidelines for pulmonary embolism, based on echocardiographic signs of right ventricular pressure overload, including interventricular septal flattening (D-shaped left ventricle) [2]. In this study, resolution of RV dysfunction was operationally defined as normalisation of interventricular septal configuration, evidenced by the disappearance of the D-shaped left ventricle on echocardiography.

In addition, exploratory mechanistic outcomes were assessed to characterise the sensitivity of different viscoelastic assays to tPA, based on changes in lysis-related parameters across tests at multiple time points. Finally, an LT cut-off was determined to identify the threshold above which clinically relevant changes in maximum clot firmness (MCF) were unlikely to occur.

### Sample size and randomisation

Randomisation followed a computer-generated sequence in a parallel-group design, with consecutive enrolment of eligible participants. Given the feasibility-oriented nature of the study and the lack of data from previous comparable studies, no formal power-based sample size calculation was performed, nor was a target allocation ratio prespecified, as the study was not designed for formal hypothesis testing. Randomisation was implemented to support feasibility assessment rather than to test treatment effects; therefore, imbalances in group sizes arising from the randomisation process were considered acceptable. Group assignment strictly followed the randomised sequence. Due to the nature of the intervention, blinding was not performed. The study period was planned for three years, including a one-year follow-up period.

### Statistical analysis

Baseline and demographic data are presented as median (interquartile range, IQR) due to the small sample size. No formal assessment of normality was performed; therefore, all analyses were conducted using non-parametric methods. Between-group comparisons were performed using the Mann–Whitney U test for continuous variables and Fisher’s exact test for categorical variables. Paired lysis time measurements obtained from different tests within the same participants across multiple time points were analysed using the Friedman test, followed by Dunn’s post hoc test when the Friedman test indicated statistical significance. Receiver operating characteristic (ROC) analysis was used to assess the discriminatory performance of lysis time (LT) for predicting relevant changes in maximum clot firmness (MCF). Analyses were primarily performed using an MCF change threshold of ≥ 85%, with additional exploratory analyses at ≥ 80% and ≥ 90% to evaluate robustness. The area under the ROC curve (AUC) was calculated together with its 95% confidence interval. A candidate LT cut-off was identified using the Youden index. In addition, sensitivity and specificity were calculated for clinically motivated LT thresholds (1800s and 2400 s) to contextualise the data-driven cut-off and to explore clinically relevant trade-offs between sensitivity and specificity. All analyses were exploratory in nature, consistent with the feasibility design of the study.

Graphs and visualisations were created using licensed GraphPad Prism (version 10.6.1) and BioRender.

## Results

### Participant flow

A total of 33 patients were screened; after exclusions (6 low- or intermediate-low risk, 2 with active bleeding, 1 with head trauma, 2 without consent), 22 remained and were randomised: 8 to the CG and 14 to the VGG.

**Fig. 2:**
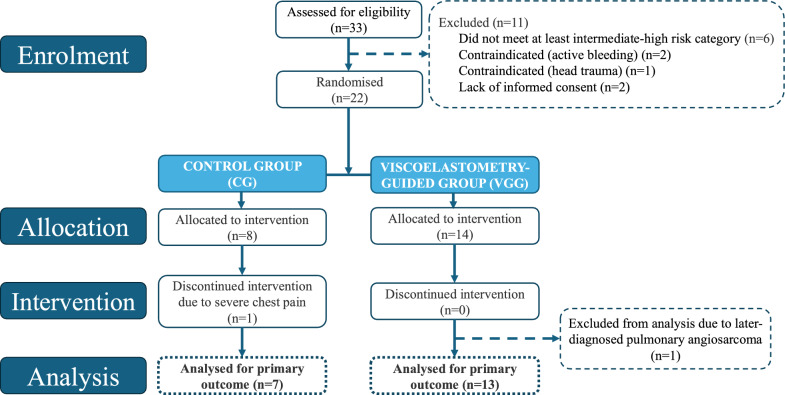
Participant flow diagram: of *33 eligible patients, 11 were excluded* (6 intermediate-low or low risk; 2 with active bleeding; 1 with head trauma, 2 lacking consent). The remaining *22 were randomised: 8 to the CG and 14 to the VGG*. In the CG, one intervention was stopped due to severe chest pain; all VGG procedures were completed. In the VGG, one patient later diagnosed with pulmonary angiosarcoma and one patient with pre-existing pulmonary hypertension were excluded from the analysis. The final analysis included *7 CG* and *12 VGG patients*

In the CG, systemic thrombolysis was discontinued in one patient due to severe chest pain. In the VGG, one patient later diagnosed with pulmonary angiosarcoma and one patient with pre-existing pulmonary hypertension were excluded from the analysis. Consequently, 7 CG and 12 VGG patients were included in the final analysis (Fig. 2).

### Recruitment

Recruitment was conducted at the Central ICU, Department of Anesthesiology and Intensive Therapy, Semmelweis University (Budapest, Hungary), between December 2021 and September 2023. Adults with confirmed acute PE by CTPA referred for potential intensive care were screened for thrombolysis eligibility, mostly admitted from the Department of Emergency Medicine.

### Baseline data

Table 1 presents patients’ demographic and baseline data, including early mortality risk scores (APACHE-II, PESI, sPESI, and CPES) [24], initial high-sensitivity troponin T and NT-proBNP levels. The detailed medical histories are provided in the Supplementary Results Table S3 (CG) and Table S4 (VGG).

**Table 1:** Baseline data: all continuous variables were expressed as median and interquartile range (IQR), and compared with the Mann–Whitney U test

	Control group (CG)	Viscoelastometry-guided group (VGG)	P value
Number of analysed patients	7	12	
Female/male	3/4	4/8	> 0.99
Age (year; median (IQR))	63.61 (60.56–70.86)	53.75 (49.84–67.38)	0.06
BMI (kg/m^2^; median (IQR))	29.41 (26.42–32.87)	29.56 (27.31–34.95)	0.83
BSA (m^2^; median (IQR))	1.97 (1.94–2.09)	2.06 (1.87–2.21)	0.53
APACHE-II (median (IQR))	11 (6–15)	9.5 (7.25–13.75)	0.85
hs Troponin T (ng/L) median (IQR)	121 (70–159)	91 (44–196)	0.44
NT-proBNP (pg/mL) median (IQR)	4502 (540–5392)	1976 (365–5491)	0.90
PE risk stratification for mortality, n			
Intermediate high-risk	2	5	0.65
High risk	5	7	
PESI	94 (87–154)	101 (77–119)	0.46
sPESI risk category, n			
Low	1	1	> 0.99
High	6	11	
CPES score median (IQR)	5 (5–6)	5 (5–6)	0.51

Categorical variables were compared using Fisher’s exact test. No significant group difference was found. (**BMI: body mass index**; **BSA**: *body surface area*; **APACHE-II**: *Acute Physiology and Chronic Health Evaluation II*; **PE**: *pulmonary embolism*; **PESI**: *Pulmonary Embolism Severity Index;* **sPESI**: *simplified Pulmonary Embolism Severity Index*; **CPES**: *Composite Pulmonary Embolism Shock Score)*

### Outcomes

#### Primary outcome: feasibility

A total of 95 h of viscoelastic sampling were completed out of the planned 100 h, corresponding to 95% adherence to the sampling protocol. During these 95 h, 632 viscoelastic tests were performed out of the planned 665 (95%), of which only five tests were excluded due to measurement error (0.8%). *Table S5* in the Supplementary Results summarises viscoelastic testing-guided dose adjustment decisions, with protocol deviations occurring in 23% of decision points. Among the 95 intervention points, 38 dose modifications (40%) were performed, comprising 26 reductions (68% of modifications) and 12 increases (32%) (see Table S6 in the Supplementary Results).

During the treatment phase, echocardiographic follow-up was successfully archived in the Picture Archiving and Communication System (PACS) for 38 of the 48 planned assessments, corresponding to a completion rate of 79.2%. This adherence falls within the predefined feasibility range, corresponding to acceptable adherence with potential minor modifications.

#### Secondary outcome 1: safety

TPA tests showed no fibrinolysis resistance before or during the thrombolysis.

**Fig. 3:**
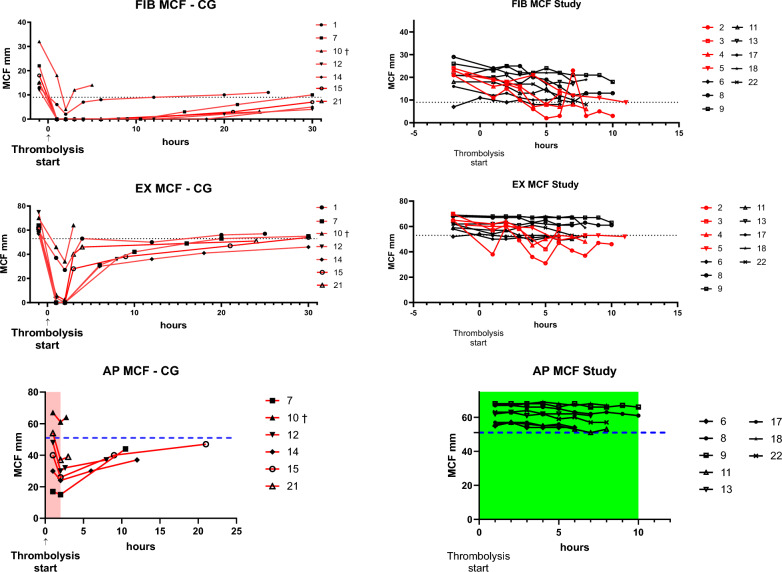
MCF values in EX, FIB, and AP tests: in the *FIB test*, all CG patients (*100%*) had MCF < 9 mm (the lower normal threshold; indicated by the dashed line), *71.4% reached 0 mm* with delayed normalisation up to 30 h. In the VGG, *none reached 0 mm*, and only *41.7%* fell below this threshold, while all of these patients received fibrinogen. The EX test revealed clear effects of fibrinogen deficiency in the CG, while in the VGG, the majority of values remained within normal limits. In the AP test, all but one CG patient dropped below normal, while none did in the VGG

In the CG, all patients (100%) had FIB MCF values below 9 mm, with 5 of 7 (71.43%) reaching 0 mm, indicating absent clot formation for up to 8 h post-thrombolysis (Fig. 3). Normalisation was slow, lasting up to 30 h. In the VGG, no patient reached 0 mm (0%), and only 5 of 12 (42%) fell below 9 mm; all received fibrinogen supplementation.

The EX test also revealed fibrinogen deficiency. In the CG, hypofibrinogenemia and coagulopathy persisted for over 30 h, whereas most VGG patients maintained normal MCF values throughout.

Similarly, in the AP test, all but one CG patient fell below the normal range, whereas all the VGG patients were in the normal range.

**Fig. 4:**
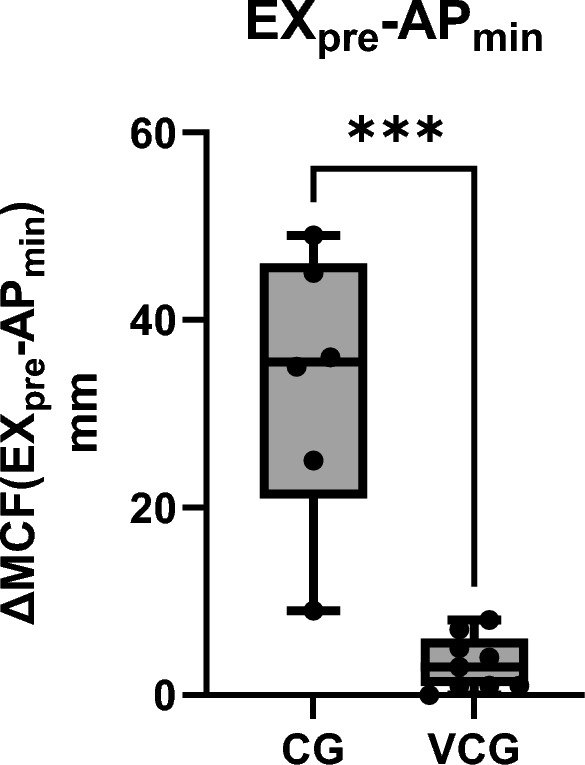
Comparison of the difference between pre-treatment EX MCF and minimum AP MCF values (EX_pre – AP_min) in the control group (CG) and the viscoelastometry-guided group (VGG). The Mann–Whitney U test revealed a significant difference between groups (p = 0.0004). Median differences were 35.5 mm in the CG and 2.0 mm in the VGG

The Mann–Whitney U test comparing the difference between pre-treatment EX MCF and minimum AP MCF values (EX_pre – AP_min) revealed a significant difference (p = 0.0004), with median differences of 35.5 mm in the CG and 2.0 mm in the VGG (Fig. 4).

The VGG received a significantly lower rtPA dose, with a median of 32.00 mg (IQR 20.95–42.75 mg).

**Fig. 5:**
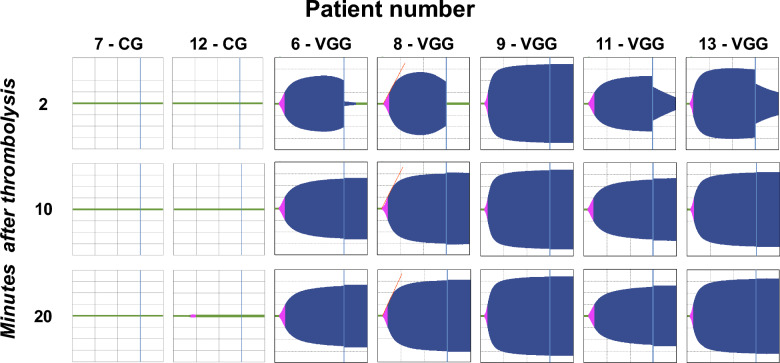
ECA tests after systemic thrombolysis: in CG, *no clot formed* even 20 min post-thrombolysis. In the VGG, clot formation was visible, with residual rtPA activity at 2 min, but no longer visible at 10 min

In selected patients, ECA tests after systemic thrombolysis showed no clot formation in the CG even 20 min post-thrombolysis. In contrast, the VGG showed clot formation, with rtPA activity persisting at 2 min but no longer visible at 10 min (Fig. 5).

Two major bleeding events (28.6%) occurred in the CG: one fatal case after resuscitation with pericardial and gastrointestinal haemorrhage, and one epistaxis-related aspiration requiring intubation and prolonged ICU stay. In the VGG, one intracranial haemorrhage (8%) occurred, possibly linked to an unreported prior collapse that had left no apparent external signs but may have contributed to the outcome.

Two-hourly ABGs showed no significant drop in haemoglobin, except in the post-resuscitation bleeding case. No occult bleeding occurred in either group.

#### Secondary outcome 2: efficacy

On the first ultrasound performed after thrombolysis, a persistent D-sign was still present in 2 of 6 CG patients. In contrast, RV dysfunction had resolved in all patients in the VGG, corresponding to efficacy rates of 66% and 100%, respectively.

As designed, systemic thrombolysis duration was prolonged in the VGG, with a median duration of 8.5 h (IQR 6.6–10.0).

#### Secondary outcome 3: additional outcomes

Paired lysis time (LT) measurements obtained from EX, FIB, and ECA tests within the same participants across multiple time points (75 × 3 paired measurements) were analysed using the Friedman test, which revealed a significant difference between tests (p < 0.0001). Post hoc pairwise comparisons with Dunn’s correction indicated the sequence of increasing sensitivity as EX < FIB < ECA (p < 0.0001 for all comparisons). The distribution of LT values from EX, FIB, and ECA tests across repeated measurements is shown in Figure S1 of the Supplementary Results.

Receiver operating characteristic (ROC) analysis was performed to evaluate the performance of LT in discriminating between different magnitudes of maximum clot firmness (MCF) change. A total of 64 paired measurements were included. For an MCF change threshold of ≥ 15%, LT showed excellent discriminatory performance, with an area under the curve (AUC) of 0.8227 (95% CI: 0.716–0.938, p < 0.0001). The corresponding Youden index was 0.6013, identifying an LT cut-off of > 2020s as the statistically optimal threshold for balancing sensitivity (74.4%) and specificity (85.7%). Sensitivity and specificity were also calculated for clinically motivated LT thresholds: at 1800 s (30 min), sensitivity was 76.7% and specificity 71.4%; at 2400 s (40 min), sensitivity was 58.1% and specificity 85.7%. Additional ROC analyses using alternative MCF change thresholds are provided in Figure S2 of the Supplementary Results.

#### Individualised rtPA dosing

After the pilot phase, the reduced-dose rtPA protocol resulted in balanced MCF values without an excessive decrease in clot firmness. This approach defined a dose range associated with these findings. Based on the dosing applied in the VGG, an initial rtPA dose of < 50 µg/h/kg (total body weight-based) or 2 mg/h/m^2^ (body surface area-based) was observed to be associated with these parameters. These values were used as reference points, with ongoing monitoring and reassessment applied to adjust the dose (see Figure S3 in the Supplementary Results). In one patient with a particularly high bleeding risk, a deviation from this protocol was applied, starting at an even lower initial dose and titrating upward until an effect was observed in the viscoelastic test of the arterial sample (see Figure S4 in the Supplementary Results).

## Discussion

### Interpretation

In systemic thrombolysis, the goal is to achieve widespread fibrinolysis to promote the dissolution of intravascular thrombus. The lungs are uniquely exposed to thrombolytic therapy because they normally receive the full cardiac output; in pulmonary embolism, this exposure may be altered by the presence of extra- or intrapulmonary shunts [25].

Several studies have explored the potential role of full-dose (100 mg) or reduced-dose thrombolysis in patients with intermediate-high risk PE, compared to anticoagulation alone with weight-adjusted low-molecular-weight heparin (LMWH), particularly considering the potential benefit of rtPA administration on both short- and long-term outcomes [4, 9, 26]. Another study found that 34.1% of intermediate-risk PE patients had normotensive shock, highlighting the need for effective intervention [27]. Current European guidelines from the European Society of Cardiology emphasise structured risk stratification and largely restrict thrombolysis to high-risk patients, whereas more recent North American guidelines adopt a more flexible, phenotype-based approach that supports individualised decision-making, particularly in intermediate-risk cases [2, 28]. Thrombolysis is inherently associated with a high risk of significant bleeding complications [5]. To mitigate this risk, studies have used fixed low rtPA doses (half or one-quarter of 100 mg) over a predefined, prolonged infusion period of 3–6 h. In one study, persistent haemodynamic instability led to repeat dosing, despite the 2019 ESC guideline advising against repeated systemic thrombolysis due to bleeding risk, especially intracranial haemorrhage [2, 10]. No major or intracranial bleeding occurred with quarter-dose rtPA (25 mg) [10, 13, 29, 30]. In a larger study, half-dose rtPA (50 mg) in intermediate-risk PE caused moderate-to-major bleeding in about 11% of patients [14]. Previous trials with echocardiographic monitoring showed immediate and significant RV improvement after thrombolysis and modest long-term functional gains [31]. The ESC’s multicentre FOCUS study found two-year incidences of 2.3% for chronic thromboembolic pulmonary hypertension (CTEPH) and 16% for post-PE impairment (PPEI). Both mainly affected intermediate- or low-risk patients treated with anticoagulation alone. PPEI was associated with a 31% rehospitalisation rate during follow-up [32].

Viscoelastic haemostatic tests are now widely used for real-time monitoring of coagulation, and may also prove essential in managing PE and thrombolysis. Early detection of rtPA-induced hyperfibrinolysis and hypofibrinogenemia may be pivotal in preventing major haemorrhagic complications. The third-generation viscoelastic tests provided by the ClotPro® system offer a broad spectrum of reagents (EX, IN, FIB, TPA, AP, RVV, and ECA) for the comprehensive assessment of real-time alterations within the coagulation cascade [33].

The ECA test identified hyperfibrinolysis in significantly more cases and at an earlier stage than the EX and IN tests. The absence of calcium in the ECA test leads to reduced activation of calcium-dependent pathways, including factor XIII (FXIII), which is essential for fibrin clot stabilisation, and thrombin-activatable fibrinolysis inhibitor (TAFI), which inhibits fibrinolysis [34–37].

The AP test contains tissue factor as an activator, polybrene as a heparin inhibitor, and aprotinin to suppress fibrinolysis induced by tPA, thereby allowing the evaluation of the total residual haemostatic capacity of the blood [38].

These considerations led to our clinical trial, the results of which are presented below.

In the following, we discuss the feasibility of the study procedures, including protocol adherence for arterial blood sampling, viscoelastic testing, dose adjustment decisions, and echocardiographic follow-up. Protocol adherence for sampling and viscoelastic testing was high, supporting the feasibility of the approach, although some aspects (e.g. dose adjustment decisions and echocardiographic follow-up) may benefit from simplification.

Dose adjustments were frequently required, highlighting the need for close, preferably hourly, monitoring to ensure safe and effective thrombolysis. Initiating thrombolysis at a lower dose reduces the need for dose reductions; although dose escalations may be more frequent, this approach is recommended to enhance procedural safety.

Adherence to protocol-guided dose adjustment decisions fell within the “feasible with modifications” range. Our findings suggest that lysis time (LT) may represent a more reliable parameter for treatment guidance than changes in MCF, allowing clearer identification of safe and high-risk fibrinolytic ranges.

Echocardiographic follow-up was successfully archived for 79% of planned assessments, a score which also falls within the predefined feasibility range but indicates that some simplification could improve adherence. In our study of low-dose tPA therapy, no cases were observed in which right ventricular dysfunction resolved within the first 4 h, suggesting that earlier assessments may be unnecessary and that protocol simplification could enhance feasibility without compromising patient monitoring.

After evaluating procedural feasibility, we explored secondary outcomes, focusing on the safety and efficacy of the reduced-dose rtPA protocol. These analyses are exploratory due to the small sample size, highlighting the need for further investigation in larger cohorts to validate these observations.

The individualised protocol better preserved overall clot integrity and prevented prolonged coagulopathy. In the VGG, none of the patients demonstrated a decrease below the normal range in the AP test, which reflects the coagulation system’s functional potential independent of circulating tPA. Our findings suggest that the VGG protocol better preserves the functional capacity of the coagulation system during thrombolysis, supporting its potential as a safer and more controlled strategy.

As additional outcomes, our analyses confirmed that the ECA test was the most sensitive test to detect the effects of tPA in the samples. This heightened sensitivity may be explained by the test’s mechanism: activation occurs with ecarin without recalcification, rendering calcium-dependent factors, such as FXIII and TAFI, involved in fibrinolysis inactive. The FIB test ranked second in sensitivity, likely because platelet function is pharmacologically inhibited in this assay, preventing FXIII expressed on activated platelet surfaces from contributing to clot stabilisation [39]. Consequently, even low levels of tPA that are insufficient to induce fibrinolysis in the FIB test can trigger detectable clot lysis in the ECA test, allowing very sensitive detection of tPA transitioning from the venous to the arterial compartment. These findings may have implications for managing bleeding-prone lysis events, although confirmatory studies are required to establish clinical relevance.

The ROC analyses demonstrated that lysis time (LT) also reliably reflects the effect of tPA present in the samples and predicts significant changes in MCF. LT demonstrated excellent discriminatory performance with an optimal cut-off of > 2020 s. Interpreting LT in a clinically meaningful way, values below 1800 s (30 min) indicate a ‘danger zone’ of high risk for extensive clot degradation, values above 2400 s (40 min) correspond to a ‘green zone’ where the clot is largely preserved, and intermediate values define a ‘grey zone’ of moderate risk. These observations suggest that LT can serve as an early functional marker of circulating tPA activity to guide individualised interventions, although further studies are needed to validate these thresholds in different clinical contexts.

Regarding the primary outcome, it is noteworthy that all components of the study protocol were successfully implemented despite increased demands on human resources, time, and cost, as well as on equipment and the need for VET and echocardiography expertise. The secondary outcome, safety, was largely dependent on cumulative rtPA exposure, whereas the other secondary outcome, efficacy, was primarily dictated by the duration of systemic thrombolysis. Integrating these additional outcomes may also inform the design and planning of a multicentre trial.

To our knowledge, this is the first study to apply ClotPro-guided thrombolysis in patients with pulmonary embolism. While previous studies have explored fixed-dose thrombolysis strategies, our findings suggest that real-time viscoelastic guidance may allow a more individualised and potentially safer approach.

### Limitations

Limitations of this study include the relatively small sample size, inherent to its single-centre design, and the lack of blinding of healthcare providers throughout treatment and follow-up. Furthermore, given the feasibility-focused design, the limited sample size and the absence of a predefined allocation ratio and formal sample size calculation, together with the observed differences in secondary outcomes related to safety and efficacy, these findings should be interpreted as exploratory and hypothesis-generating. Accordingly, while these findings are encouraging, no definitive conclusions can be drawn, and confirmation will require a larger, multicentre, randomised interventional clinical trial.

## Conclusions

In summary, the ClotPro®-guided, prolonged, low-dose systemic thrombolysis protocol described in our study presents certain drawbacks, including increased workload and cost.

Based on the feasibility findings, a refined protocol for future studies may be proposed. Viscoelastic testing should be performed hourly, as less frequent measurements would not allow timely therapeutic adjustments. Treatment guidance should rely on lysis time (LT) rather than maximum clot firmness (MCF). Echocardiographic follow-up should begin at 6 h from the start of treatment, with subsequent assessments performed every 2 h.

The viscoelastic test-guided, echocardiography-controlled, prolonged thrombolytic protocol developed by our group has been shown to be feasible and, with minor modifications outlined above, may be applicable in future studies. Given the limited sample size, these findings should be considered hypothesis-generating. However, the protocol may offer several potential advantages, including preventing severe coagulopathy, enabling targeted fibrinogen supplementation, facilitating rapid restoration of coagulation upon termination of systemic thrombolysis, providing real-time monitoring of therapeutic efficacy through echocardiography, and significantly reducing the total rtPA dose administered.

These observations cautiously suggest that this approach may be associated with improved safety and efficacy compared with the widely used standard protocol based on the 2019 ESC guidelines. Confirmation of these findings will require large-scale, multicentre studies.

## Supplementary Information

Additional file 1.

## Data Availability

The ethical approval and the complete dataset are available upon request.

## Abbreviations

ABG: Arterial blood gas
APACHE-II: Acute Physiology and Chronic Health Evaluation-II
BMI: Body mass index
BSA: Body surface area
CG: Control group
CPES: Composite Pulmonary Embolism Shock score
CTEPH: Chronic thromboembolic pulmonary hypertension
CTPA: Computed tomographic pulmonary angiography
ECG: Electrocardiogram
ESC: European Society of Cardiology
FDP: Fibrin degradation product
FXIII: Factor XIII
ICU: Intensive care unit
IQR: Interquartile range
LLM: Large language model
LMWH: Low-molecular-weight heparin
LT: Lysis time
MCF: Maximum clot firmness
ML: Maximum lysis
PE: Pulmonary embolism
PESI: Pulmonary Embolism Severity Index
PPEI: Post-pulmonary embolism impairment
rtPA: Recombinant tissue plasminogen activator
RV: Right ventricle
SD: Standard deviation
sPESI: Simplified Pulmonary Embolism Severity Index
TAFI: Thrombin-activatable fibrinolysis inhibitor
tPA: Tissue plasminogen activator
TTE: Transthoracic echocardiography
VET: Viscoelastic test
VGG: Viscoelastometry-guided group
