# Supplementary material for: Personalised viscoelastometry-guided systemic thrombolysis for high- and intermediate-high-risk acute pulmonary embolism in the ICU: a single-centre randomised controlled interventional feasibility trial
Source: Intensive Care Med Exp. 2026 Apr 29;14:57. doi: 10.1186/s40635-026-00903-7 (PMC13129181; doi:10.1186/s40635-026-00903-7)
Supplement: Supplementary file 1 — Additional file 1. [file 40635_2026_903_MOESM1_ESM.docx]

Supplementary Material

**Methods**

| **Early mortality risk** | **Haemodynamic instability** | **Clinical parameters of PE severity and/or comorbidity (PESI class III–V or sPESI ≥1)** | **RV dysfunction on TTE or CTPA** | **Elevated cardiac troponin levels** |
| --- | --- | --- | --- | --- |
| **High** | **+** | **(+)*** | **+** | **(+)** |
| **Intermediate- high** | **–** | **+** | **+** | **+** |
| **Intermediate- low** | **–** | **+** | **One (or none) positive** | **– / +** |
| **Low** | **–** | **–** | **–** | **Assessment optional; if assessed, negative** |

Table S1.: Early mortality risk stratification in acute pulmonary embolism (ESC 2019) [[2]](https://paperpile.com/c/aIjjt1/0F3K)

| **Category** | **Contraindication** |
| --- | --- |
| **Absolute contraindications** | History of haemorrhagic stroke or stroke of unknown origin |
|  | Ischaemic stroke within the previous 6 months |
|  | Central nervous system neoplasm |
|  | Major trauma, surgery, or head injury within the previous 3 weeks |
|  | Bleeding diathesis |
|  | Active bleeding |
| **Relative contraindications** | Transient ischaemic attack within the previous 6 months |
|  | Oral anticoagulation |
|  | Pregnancy or first postpartum week |
|  | Non-compressible puncture sites |
|  | Traumatic resuscitation |
|  | Refractory hypertension (systolic blood pressure >180 mmHg) |
|  | Advanced liver disease |
|  | Infective endocarditis |
|  | Active peptic ulcer |

Table S2.: Contraindications to systemic thrombolysis (ESC 2019) [[2]](https://paperpile.com/c/aIjjt1/0F3K)

**Results**


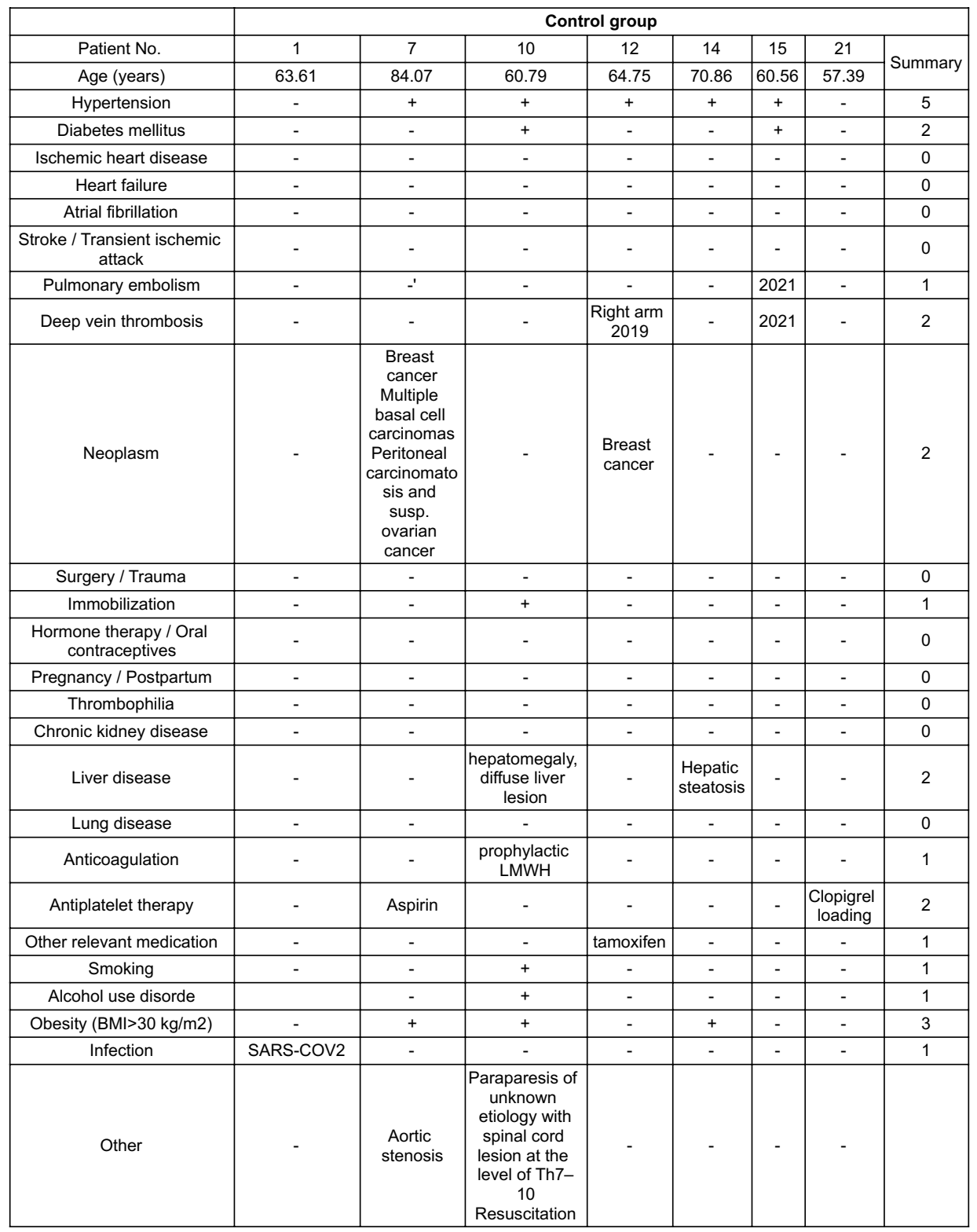


Table S3: Comorbidities of the control group (CG): Presence of individual specific comorbidities in the included patients, as well as their overall prevalence.


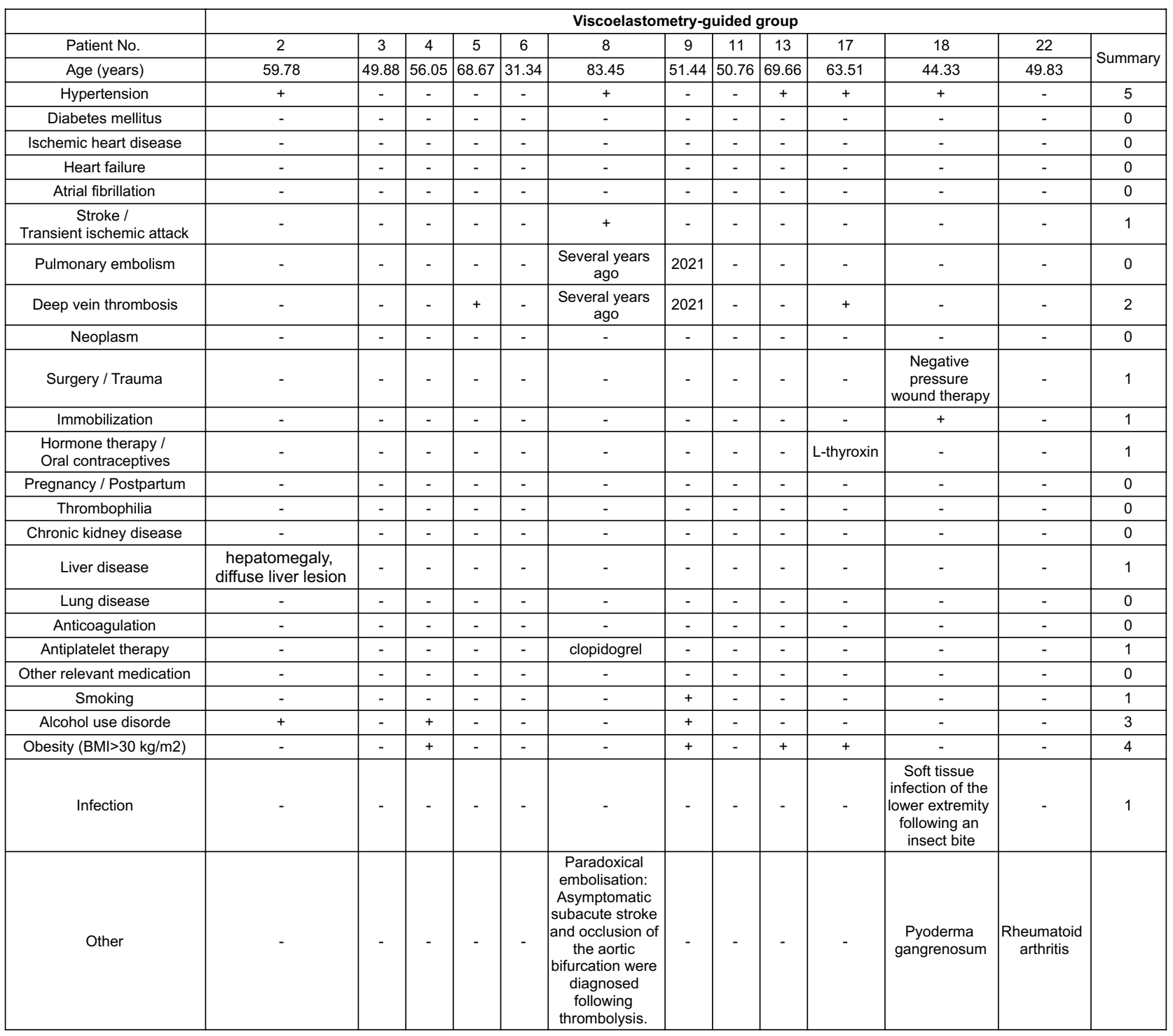


Table S4: Comorbidities of the Viscoelastometry-guided group (VGG): Presence of individual specific comorbidities in the included patients, as well as their overall prevalence.

| **Dose adjustment category** | **n** | **%** |
| --- | --- | --- |
| **Total viscoelastometry-guided decision points** | **87** | **100** |
| Inappropriate dose reduction | 7 | 8.05 |
| Inappropriate dose escalation | 5 | 5.75 |
| Dose escalation indicated but not performed | 2 | 2.30 |
| Dose reduction indicated but not performed | 6 | 6.90 |
| Appropriate dose escalation | 4 | 4.60 |
| Appropriate dose reduction | 19 | 21.84 |
| **Overall protocol deviations** | **20** | **23%** |

Table S5.: Viscoelastic testing–guided dose adjustment decisions, with protocol deviations

| **Study phase / category** | **n** | **%** |
| --- | --- | --- |
| **Pilot phase – total intervention points** | **31** | **100** |
| Dose reduction (pilot) | 13 | 41.9 |
| Dose increase (pilot) | 2 | 6.5 |
| **Final phase – total intervention points** | **64** | **100** |
| Dose reduction (final) | 13 | 20.3 |
| Dose increase (final) | 10 | 15.6 |
| **Overall intervention points** | **95** | **100** |
| **Total dose adjustments** | **38** | **40.0** |
| └ Total dose increases | 12 | 31.6 |
| └ Total dose reductions | 26 | 68.4 |

Table S6.: Viscoelastic testing–guided dose adjustments across study phases, demonstrating a lower proportion of required interventions during the final phase, consistent with lower initial tPA doses and improved protocol implementation over time.


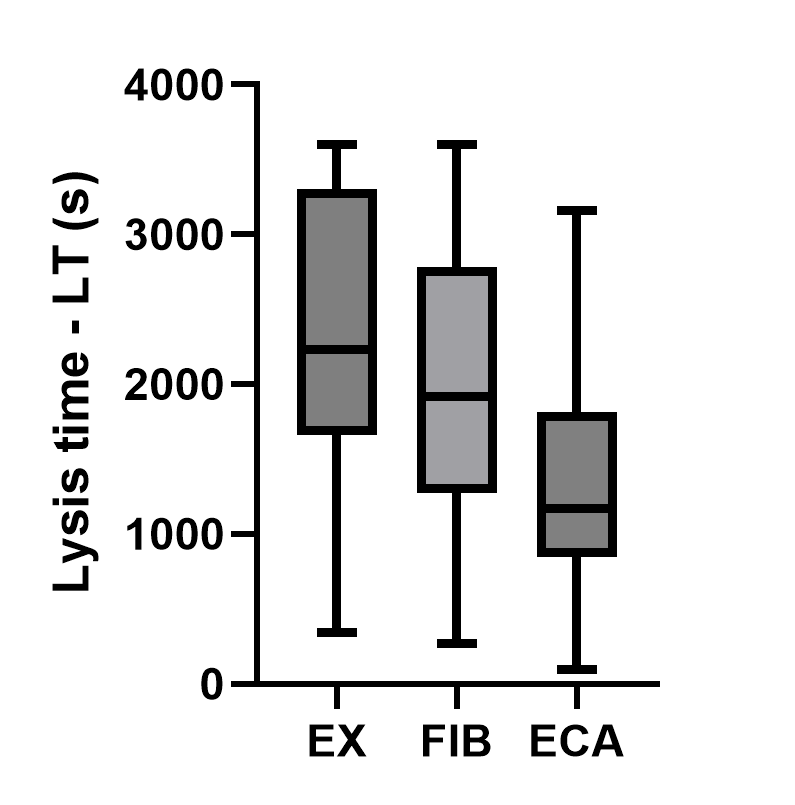


*Figure S1*. Box-and-whisker plots showing lysis time (LT) values obtained from EX, FIB, and ECA tests across multiple time points (75 × 3 paired measurements).

Only measurements where at least one test had a detectable LT (ML >50%) were included. Non-measurable LT values were assigned a value of 3600 s. Boxes represent the interquartile range with median values indicated, and whiskers denote 1.5×IQR. Statistical comparison using the Friedman test revealed a significant difference between tests (p < 0.0001), and post hoc pairwise comparisons with Dunn’s correction indicated increasing sensitivity as EX < FIB < ECA (p < 0.0001 for all comparisons).


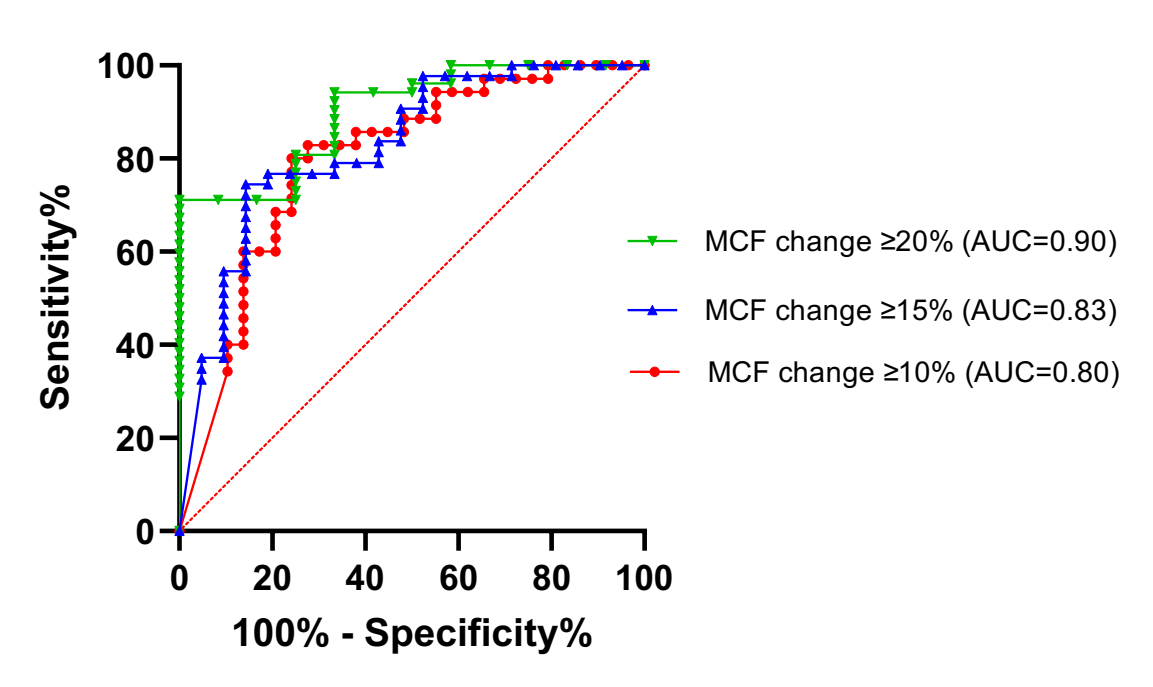


*Figure S2*. ROC curves of lysis time (LT) for predicting different magnitudes of MCF change (≥20%, ≥15%, and ≥10%).

LT was evaluated at three predefined MCF change thresholds. The corresponding AUCs were 0.90, 0.83, and 0.80, with 95% confidence intervals of 0.82–0.98, 0.72–0.94, and 0.68–0.91, respectively (p < 0.0001 for all analyses). Patient distributions by MCF change threshold (≥ threshold vs. < threshold) were 12–52, 21–43, and 29–35. Optimal cut-off values determined by the Youden index were >1924, >2020, and >2020, yielding Youden indices of 0.71, 0.60, and 0.56. These findings demonstrate consistent discriminatory performance of LT across multiple MCF change thresholds, supporting the robustness of the statistical analysis.


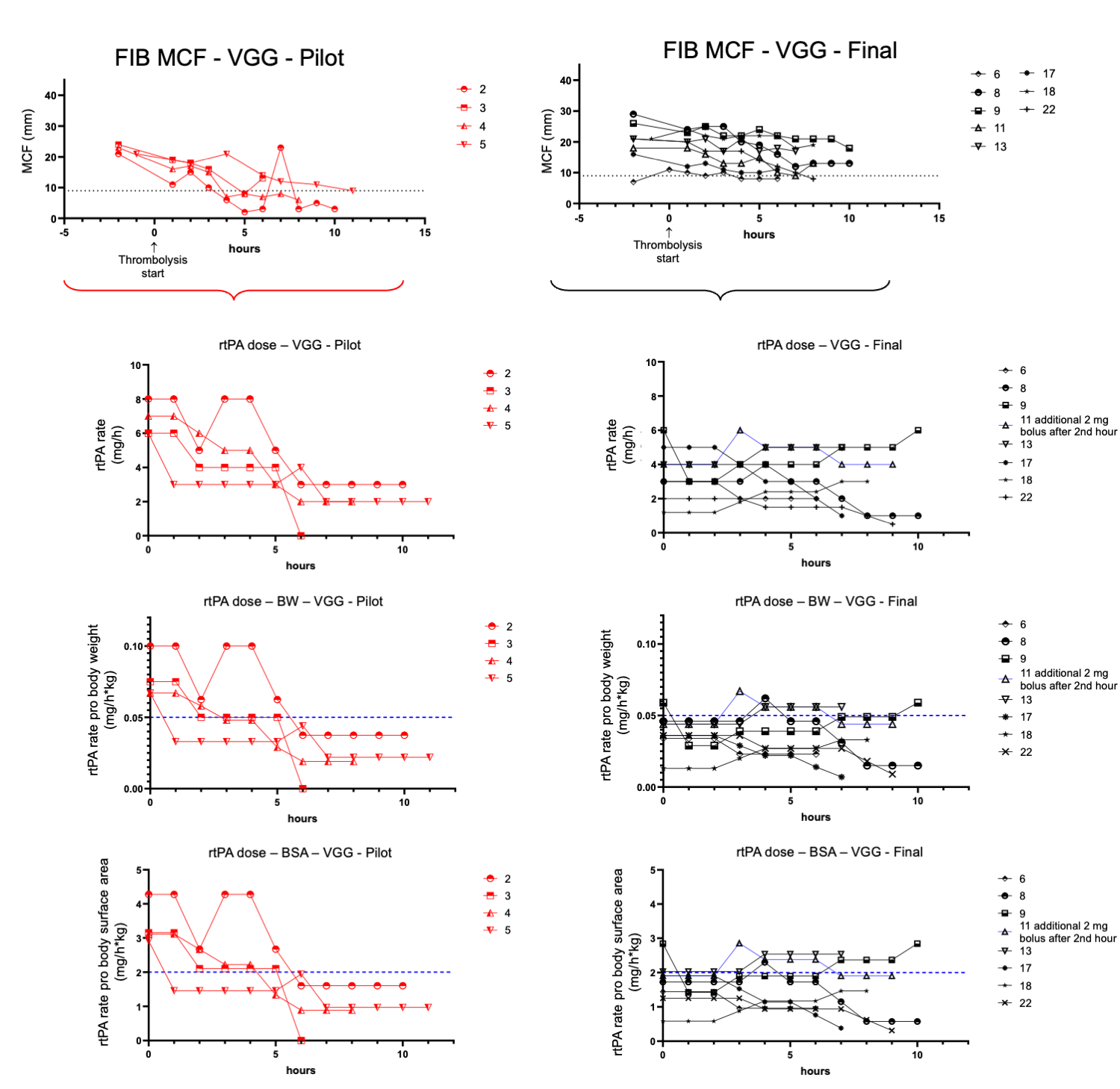


*Figure S3.* Hourly FIB MCF values and absolute, weight- and BSA-standardised rtPA doses in VGG.

After the pilot phase, a reduced-dose rtPA protocol based on body weight (BW) and body surface area (BSA) yielded balanced maximum clot firmness (MCF) values without causing excessive reduction in clot firmness. FIB MCF levels can be maintained within the normal range if the rtPA bolus and initial infusion rate do not exceed 50 µg/kg/h or 2 mg/m²/h, as indicated by the blue dashed lines, with subsequent adjustments guided by follow-up measurements.


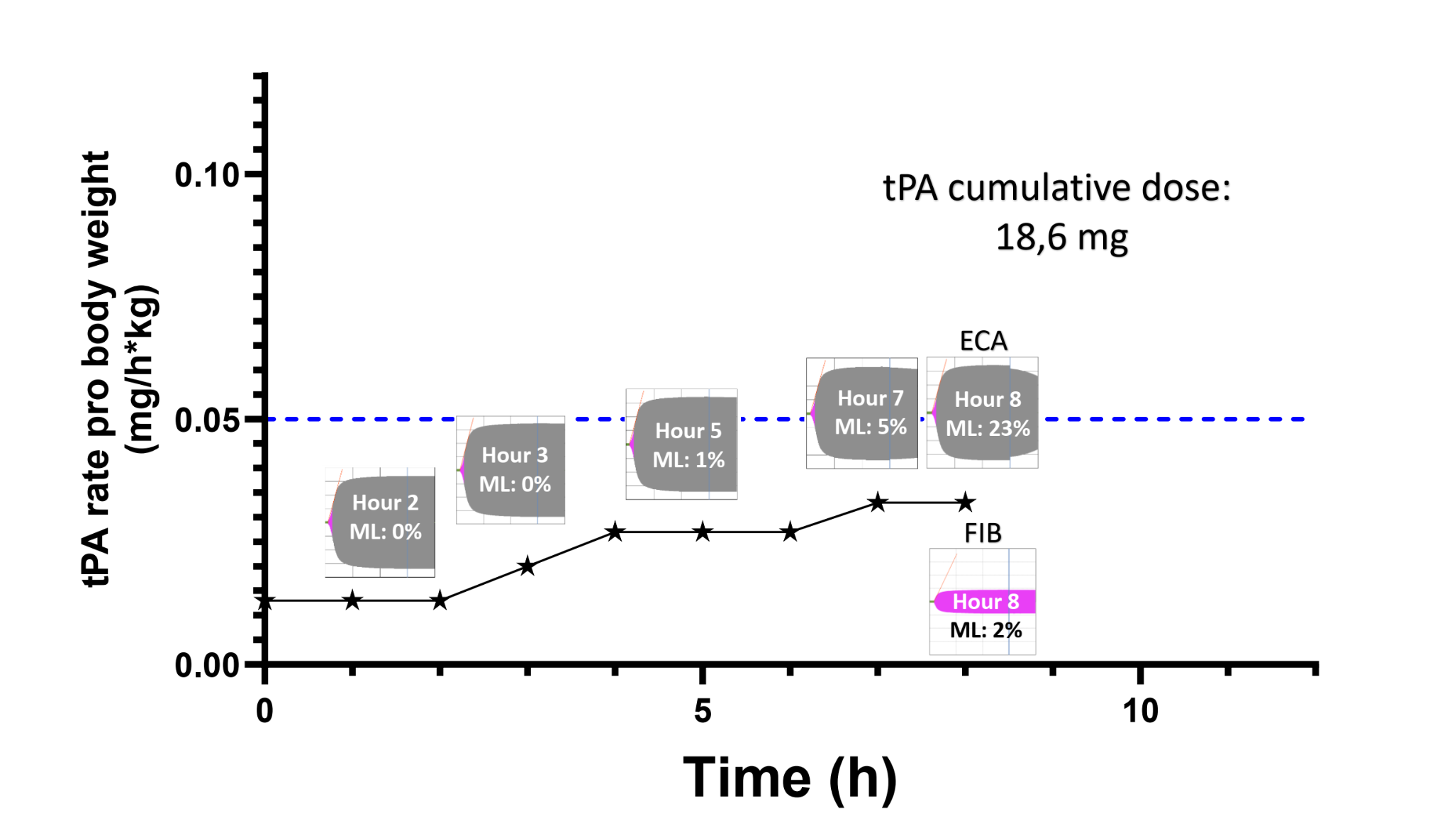


*Figure S4.* Case example of individualised rtPA dose titration in a high bleeding risk patient

A 44-year-old patient (Patient 18) was admitted from the operating room following surgical debridement and initiation of vacuum therapy. Intraoperative hypoxia and hemodynamic instability prompted CT angiography, revealing pulmonary embolism. Due to the elevated bleeding risk, thrombolytic dosing was carefully titrated upward from a very low initial dose (13.3 µg/kg/h) until fibrinolysis was detected only by the ECA test, the most sensitive assay for fibrinolysis (see Figure S1 in the Supplementary Results for further explanation). At hour 7, at a dose of 33.3 µg/kg/h, fibrinolysis was evident in the ECA test, while no fibrinolysis was detectable in the FIB test. This targeted, assay-guided titration enabled effective thrombolysis without bleeding complications despite the extensive postoperative wound. Subsequently, the echocardiographic D-sign resolved at hour 8, and thrombolysis was discontinued.
